# Impact of COVID-19 and system recovery in delivering healthcare to people with multiple sclerosis: a population-based Study

**DOI:** 10.1007/s10072-023-07052-9

**Published:** 2023-09-06

**Authors:** Giuseppina Affinito, Ugo Trama, Laura Palumbo, Maria Grazia Fumo, Roberta Giordana, Massimo Di Gennaro, Maria Triassi, Roberta Lanzillo, Vincenzo Brescia Morra, Raffaele Palladino, Marcello Moccia

**Affiliations:** 1 Department of Public Health, University of Naples Federico II, Via Sergio Pansini 5, 80131 Naples, Italy; 2 Drug Policy and Devices Unit, Regione Campania Health Department, Naples, Italy; 3 Regional Healthcare Society (So.Re.Sa), Naples, Italy; 4 Innovation and Data Analytics (So.Re.Sa), Naples, Italy; 5 Department of Neuroscience, Reproductive Sciences and Odontostomatology, Federico II University of Naples, Naples, Italy; 6 Multiple Sclerosis Unit, Policlinico Federico II University Hospital, Naples, Italy; 7 Department of Primary Care and Public Health, Imperial College, London, UK; 8 Department of Molecular Medicine and Medical Biotechnology, Federico II University, Naples, Italy

**Keywords:** COVID-19, Multiple sclerosis, Healthcare, Recovery, DMTs, Epidemiology, Pandemic

## Abstract

**Background:**

COVID-19 pandemic has affected the management of multiple sclerosis (MS).

**Objective:**

To explore the impact of COVID-19 on healthcare delivery to people with MS and the subsequent recovery of the system.

**Methods:**

In this population-based study in the Campania Region (Italy), we included people with MS across pre-COVID-19, lockdown, pre-vaccination, and vaccination periods. Differences in continuous outcomes between periods were explored using linear mixed models (annualized hospitalization rate (AHR) and adherence measured as medication possession ratio (MPR)). Differences in disease-modifying treatment (DMT) prescription rates (first DMT prescription, any DMT switch, switch from platform to highly effective DMT, and combination of first DMT prescription and any DMT switch) were assessed using an interrupted time series design.

**Results:**

Compared with pre-COVID-19, AHR decreased during the lockdown (Coeff = 0.64;95%CI = -0.69, -0.59; *p* < 0.01), and remained lower during pre-vaccination and vaccination periods. Adherence decreased during pre-vaccination (Coeff = -0.04;95%CI = -0.05, -0.03; *p* < 0.01) and vaccination periods (Coeff = -0.07;95%CI = -0.08, -0.07; *p* < 0.01). After the lockdown, there was an increase in any DMT switch (IRR 2.05 95%CI 1.38,3.05; *p* < 0.01), in switch from platform to highly effective DMTs (IRR 4.45;95%CI 2.48,8.26; *p* < 0.01) and in first DMT prescriptions (IRR 2.48;95%CI 1.64,3.74; *p* < 0.01).

**Conclusions:**

DMT prescriptions quickly returned to pre-pandemic levels, reflecting good health system recovery. However, adherence has remained lower than the past, as from suboptimal care. Assessing long-term COVID-19 impact on MS healthcare is warranted.

## Introduction

Coronavirus disease 2019 (COVID-19) was first identified in December 2019 in the city of Wuhan, China, and rapidly became a pandemic, with 6,636,278 deaths out of 646,266,987 confirmed cases worldwide, as of December 2022 [1]. Italy was the first European country affected by COVID-19 with 24,709,404 confirmed total cases and 182,419 deaths to date [2]. In the initial phase of the pandemic (e.g., emergency phase, the great lockdown), massive disruptions involved healthcare systems all over the world, leading to a fast reorganization of people, structures, and devices. All the non-urgent clinical activities, such as follow-ups, treatments, and tests for chronic diseases, were suspended [3]. Moreover, the outbreak of COVID-19 has led to increased workload, psychological distress, and infection risks among medical staff causing a drastic decrease in its recovery [4–6]. COVID-19 pandemic has had multiple waves of contagion (e.g., autumn 2020 and spring 2021), and has only improved across 2021 thanks to mass vaccination campaign, which has proven effective at reducing both risk and severity of infection, with some caveats in immunocompromised patients.

As such, the COVID-19 pandemic has brought challenges to the healthcare management of people with multiple sclerosis (PwMS). Indeed, PwMS require multidisciplinary management and access to a broad range of services, including regular specialty examinations, diagnostic tests, rehabilitation, psychological support, social care and inclusion services [7–10]. In addition, PwMS need long-term treatment with immunomodulatory and immunosuppressive disease-modifying therapies (DMT), to decrease the relapse rate and potentially prevent disability accumulation. Still, very few studies have quantified the impact of COVID-19 on healthcare delivery to people with MS [11, 12], and none has evaluated whether and to what extent activities have resumed to pre-pandemic levels.

Therefore, in our population-based study conducted in the Campania Region (South Italy), we aimed to evaluate the impact of the COVID-19 pandemic (i.e., across its different phases and after vaccination campaign) and the recovery of the healthcare system in delivering services to PwMS.

## Methods

### Study design

This is a population-based study, obtained from the retrospective analysis of routinely collected healthcare data of individuals with MS resident in the Campania Region (South Italy), from 2015 to 2021 (5,624,420 inhabitants).

The study was approved by the Federico II Ethics Committee (332/21). All patients signed informed consent authorizing the use of anonymized, routinely collected healthcare data, in line with data protection regulation (GDPR EU2016/679). The study was performed in accordance with good clinical practice and Declaration of Helsinki.

### Study population

The dataset was created by merging different data sources of the Campania Region [13]. Following validation study [13], the cohort comprised all residents in the Campania Region who had at least one MS-specific record, from 2015 to 2021, in any of the routinely-collected healthcare databases, including:

1. Hospital Discharge Record database, which included all admissions in the study period with ICD-9 CM codes of MS in discharge diagnoses.
2. Regional Drug Prescription database, which included all MS-specific DMTs prescribed in the study period (e.g., alemtuzumab, cladribine, dimethyl fumarate, fingolimod, glatiramer acetate, interferon Beta-1a, interferon Beta-1b, natalizumab, ocrelizumab, peg-Interferon Beta-1a, teriflunomide).
3. Outpatient database with exemption code for MS.

The case-identification algorithm was validated towards a clinical registry, and showed 99.0% sensitivity, with only 2.7% of cases remaining undetected [13]. From the datasets, individuals with a diagnosis of MS not resident in the Campania Region were excluded. Data was fully anonymized by the Campania Region Healthcare Regulatory Society (So.Re.Sa.) before releasing the datasets.

#### COVID-19 timeline

The first recorded case of COVID-19 in the Campania Region dates to 26 February 2020. Starting in early-March 2020, activities within hospitals underwent a rapid re-organisation suspending all non-urgent clinical activities. From mid-May 2020, elective and specialty outpatient activities were resumed. Finally, in January 2021 the vaccination campaign began, with priority to healthcare workers and at-risk groups, including PwMS. As of December 2021, there have been 2,368,439 confirmed total COVID-19 cases and 11,423 COVID-19 related deaths.

Thus, in the study, we identified four-time periods:

- Pre-COVID-19 Period (as reference): from 1^st^ January 2015 to 29^th^ February 2020
- Lockdown Period: from 1^st^ March 2020 to 31^st^ May 2020
- Pre-Vaccination Period: from 1^st^ Jun 2020 to 31^st^ December 2020
- Vaccination Period: from 1^st^ January 2021 to 31^st^ December 2021

#### Demographic, clinical and treatment variables

Demographic information were year of birth and sex.

The Charlson Comorbidity Index was computed in patients with hospital discharge records, by assigning different weights to comorbidities reported in primary and secondary discharge diagnoses; the Charlson Comorbidity Index provides the risk of death from comorbidities [14] and has already been applied to MS studies [15].

DMT prescriptions were collected and based on regulatory approval. DMTs were further classified into platform (teriflunomide, interferon beta, glatiramer acetate, dimethyl fumarate) and highly effective (fingolimod, alemtuzumab, cladribine, ocrelizumab, natalizumab). Also, based on our previously validated algorithm, we identified newly diagnosed patients and respective first DMT [16].

Considering that the same individual might have been treated with different DMTs over time, or with the same DMT over different COVID-19 phases, we used individual treatment periods (ITPs) as unit for the analyses clustered at the individual level.

We also evaluated the following outcomes related to prescriptions: any DMT switch; switch from platform to highly effective DMT; and combination of first DMT prescription and any DMT switch. For each modality of new DMT prescription (first DMT prescription, any DMT switch, switch from platform to highly effective DMT, and combination of first DMT prescription and any DMT switch), we calculated the rate of prescription as the number of patients with new DMT prescription per month, divided by the total number of patients.

Adherence was estimated using the medication possession ratio (MPR) (MPR = (medication supply obtained during follow-up period/medication supply expected during the follow-up period)) [17].

#### Healthcare resource utilization and costs

Healthcare resource utilization was extracted from Campania Region datasets (i.e., hospital discharge records, regional prescribing database, and outpatient services). Healthcare resource utilization included MS-related and non-MS-related hospital admissions, which were classified based on the main discharge diagnosis. The number of hospital admissions was then reported on annual basis (annualized hospitalization rates (general AHR and MS AHR)).

Direct healthcare costs were derived from regional datasets, referred to corresponding healthcare resource utilization, and inflated to the most recent values (2021) (https://www.soresa.it/), to avoid variations in price per unit of service through different years.

### Statistical analysis

Study variables were described as mean (standard deviation), median (range), or number (percent), as appropriate. Differences in continuous outcomes between periods (Pre-lockdown (as reference), Lockdown, Pre-Vaccination and Vaccination) were explored using linear mixed models (for AHR, costs, and MPR). Covariates were age, sex, and treatment duration. Statistical models were then run including adherence and Charlson comorbidity index (for the subgroup of patients with hospital discharge records) among covariates.

Differences in new DMT prescription rates were assessed employing an interrupted time series design using a Poisson distribution with robust standard errors accounting for heteroskedasticity across patients (partly adjusted models) [18]. Specifically, for these analyses, we divided the study period as pre-lockdown and post lockdown, considering lockdown as the intervention period. Pre- and post-vaccination periods were merged in a single post-lockdown period to allow sufficient time to switch from one treatment to another (i.e., pre-vaccination period lasted only six months which might be not sufficient for a clinical evaluation before switching to another treatment). These models provided the step change after the lockdown and the slope change over the following months, as compared with pre-lockdown period (January 2019 to March 2020). In particular, we restricted the pre-lockdown period to account for the most recent DMT prescription trend before COVID-19, and also in light of new DMTs being approved from 2019 (e.g., ocrelizumab, cladribine) [19]. Analyses were then adjusted for sex and age (fully adjusted models).

Results were reported as adjusted coefficient (Coeff), incidence rate ratio (IRR), 95% confidence intervals (95%CI), and p values, as appropriate. Results were considered statistically significant for *p* < 0.05. Statistical analyses were performed using Stata 15.0.

## Results

Out of 7,431 prevalent MS patients in the Campania Region from 2015 to 2021 [16], we included 6,097 patients(age 41.47 ± 12.42; females 64%), corresponding to 8,760 ITPs (the same individual being treated with different DMTs within the study period). We excluded 1,334 patients due to missing data in relation to demographics or other study variables. Demographic, comorbidities, treatment features of included patients are reported in Table 1.

**Table 1:** Demographic, treatment, and clinical variables

	Pre-Covid	Lockdown	Pre-Vaccination	Vaccination
*Age, years, mean (SD)*	41.91 (12.19)	45.66 (12.27)	44.92 (12.39)	45.37 (12.63)
*Sex, female (%)*	*65%*	*66%*	*66%*	*65%*
*Individual Treatment Period (N)*				
*Interferon beta 1*	*2,646*	*1,004*	*1,139*	*1,135*
*Glatiramer acetate*	773	301	366	356
*Fingolimod*	1,219	346	741	804
*Alemtuzumab*	75	*0*	1	0
*Cladribine*	9	1	11	20
*Ocrelizumab*	209	11	63	418
*Dimethyl fumarate*	1,206	682	848	989
*Natalizumab*	580	362	445	565
*Months of treatment duration, mean (SD)*	42.22 (17.92)	2.03 (0.43)	5.53 (1.11)	9.81 (2.14)
*MPR, mean (SD)*	0.98 (0.20)	1.11 (0.27)	0.99 (0.22)	0.94 (0.26)
*MPR* > *80%, number (%)*	*84%*	*93%*	*85%*	*82%*
*Charlson comorbidity index*				
*0*	2,597	27	458	1,033
*1–2*	60	0	5	23
> = *3*	2	0	0	0

### New DMT prescriptions

New DMT prescription rates, along with partly and fully adjusted results, are reported in Table 2.

**Table 2:** New DMT prescriptions

*Outcome*	*Monthly rate*		*Partly Adjusted Results*			*Fully Adjusted Results*		
	*Pre-Lockdown (Over 1000)*	*Post-Lockdown (Over 1000)*	IRR*	*p-value*	*95%CI*	IRR*	*p-value*	*95%CI*
*Any DMT switch*	*9.12*	*5.42*						
*Step change*			*2.06*	*p* < *0.01*	*(1.39; 3.06)*	*2.05*	*p* < *0.01*	*(1.30; 3.05)*
*Slope change*			*0.96*	*p* < *0.01*	*(0.93; 0.98)*	*0.95*	*p* < *0.01*	*(0.93; 0.98)*
*Switch from platform to highly effective DMT effective DMT*	*4.12*	*2.31*						
*Step change*			*4.54*	*p* < *0.01*	*(2.49; 8.29)*	*4.45*	*p* < *0.01*	*(2.48; 8.26)*
*Slope change*			*0.92*	*p* < *0.01*	*(0.88; 0.96)*	*0.92*	*p* < *0.01*	*(0.88; 0.95)*
*First DMT prescription*	*6.02*	*5.54*						
*Step change*			*2.52*	*p* < *0.01*	*(1.67; 3.79)*	*2.48*	*p* < *0.01*	*(1.64; 3.74)*
*Slope change*			*0.94*	*p* < *0.01*	*(0.91; 0.96)*	*0.94*	*p* < *0.01*	*(0.91; 0.97)*
*Combination of first DMT prescription and any DMT switch*	*15.01*	*11.02*						
*Step change*			*2.03*	*p* < *0.01*	*(1.54; 2.68)*	*2.01*	*p* < *0.01*	*(1.53; 2.66)*
*Slope change*			*0.95*	*p* < *0.01*	*(0.94; 0.97)*	*0.96*	*p* < *0.01*	*(0.94; 0.97)*

Table shows the monthly rate of new DMT prescriptions, including first DMT prescription, any DMT switch, switch from platform to highly effective DMT, and combination of first DTM prescription and any DMT. The monthly rates were calculated as the number of patients with new DMT prescription per month, divided by the total number of patients (over 1000). Differences in the DMT prescription rates were assessed employing an interrupted time series design using Poisson distribution with robust standard errors accounting for heteroskedasticity across patients. For this analysis, we restricted the pre-lockdown period from Jan 2019 to March 2020. The adjusted analyses were adjusted for sex and age. * Incidence rate ratio

**Fig. 1:**
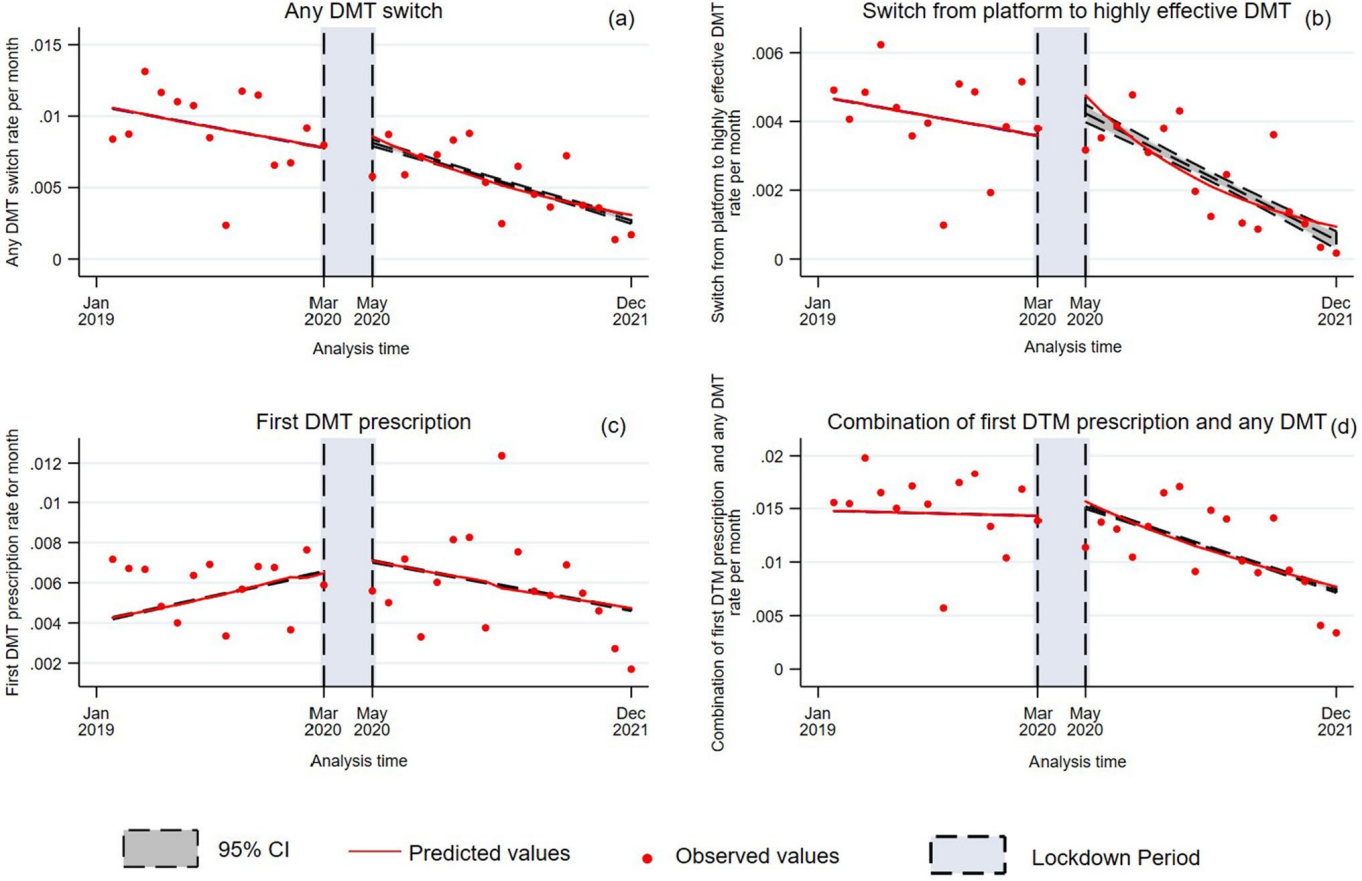
New DMT prescription rates as a function of analysis time (2019–2021). Figure shows differences in the rates of new DMT prescription (**a,** any DMT switch; **b**, switch from platform to highly effective DMT; **c**, first DMT prescription**; d**, combination of first DTM prescription and any DMT), which were assessed employing an interrupted time series design using a Poisson distribution with robust standard errors. Specifically for these analyses, we divided the study period as pre-lockdown and post lockdown, considering lockdown as the intervention period (blue shades). Monthly new DMT prescription rates (red dots) were measured as the number of patients with new DMT prescription per month, divided by the total number of patients (over 1000). Red lines show slope changes (along with 95%CI as grey shades)

After the lockdown, there was a two-fold increase in any DMT switch (step change IRR 2.05 95%CI 1.38, 3.05; *p* < 0.01), as compared with before COVID-19, which however was not sustained over time (slope change IRR 0.95; 95%CI 0.93, 0.98; *p* < 0.01) (Table 2; Fig. 1a). After the lockdown, there was a four-fold increase in switch from platform to highly effective DMTs (step change IRR 4.45; 95%CI 2.48, 8.26; *p* < 0.01), as compared with before COVID-19, which however was not sustained over time (slope change IRR 0.92; 95%CI 0.88, 0.95; *p* < 0.01) (Table 2; Fig. 1b). After the lockdown, there was a two-fold increase in first DMT prescription (step change IRR 2.48; 95%CI 1.64, 3.74; *p* < 0.01), as compared with before COVID-19, which however was not sustained over time (slope change IRR 0.94; 95%CI 0.91, 0.97; *p* < 0.01) (Table 2; Fig. 1c). After the lockdown, there was a two-fold increase in combination of first DMT prescription and any DMT prescription (IRR 2.01; 95%CI 1.53, 2.66; *p* < 0.01), as compared with before COVID-19, which however was not sustained over time (slope change 0.96; 95%CI 0.94, 0.97; *p* < 0.01) (Table 2; Fig. 1d).

### Adherence

Adherence to treatment is reported in Table 1. When compared with pre-COVID-19 period, adherence (MPR) remained similar during lockdown (Coeff = 0.06; 95%CI = 0.05,0.07; *p* < 0.01) but decreased during pre-vaccination (Coeff = -0.04; 95%CI = -0.05, -0.03; *p* < 0.01) and vaccination periods (Coeff = -0.07; 95%CI = -0.08, -0.07; *p* < 0.01).

### Healthcare resource utilization and costs

Healthcare resource utilization and costs are reported in Table 3.

**Table 3:** Healthcare resource utilization and costs

	*Pre-Covid (ref)*	*Lockdown*				*Pre-Vaccination*				*Vaccination*			
			Adjusted Results*				Adjusted Results*				Adjusted Results*		
	*Mean (SD)*	*Mean (SD)*	*Coeff*	*p-value*	*95%CI*	*Mean (SD)*	*Coeff*	*p-value*	*95%CI*	*Mean (SD)*	*Coeff*	*p-value*	*95%CI*
*Annualized hospitalization rates*	0.86 (0.97)	0.06 (0.55)	*-0.64*	*p* < *0.01*	*(-0.69; -0.59)*	0.33 (14.08)	*-0.37*	*p* < *0.01*	*(-0.41; -0.33)*	0.38 (1.01)	*-0.35*	*p* < *0.01*	*(-0.39; -0.32)*
*MS Annualized hospitalization rates*	0.79 (0.93)	0.03 (0.47)	*-0.57*	*p* < *0.01*	*(-0.62; -0.53)*	0.29 (0.99)	*-0.31*	*p* < *0.01*	*(-0.36; -0.23)*	0.34 (0.95)	*-0.29*	*p* < *0.01*	*(-0.32; -0.26)*
*Monthly Hospital admission Costs (EUR)*	40.70 (137.86)	7.71 (150.53)	*-40.19*	*p* < *0.01*	*(-48.83; -31.56)*	26.18 (179.74)	*-22.34*	*p* < *0.01*	*(-30.08; -14.59)*	29.30 (200.24)	*-22.25*	*p* < *0.01*	*(-29.35; -15.16)*
*Monthly MS hospital admission costs (EUR)*	30.65 (103.81)	1.08 (18.37)	*-35.26*	*p* < *0.01*	*(-40.93; -29.61)*	18.26 (130.08)	*-18.99*	*p* < *0.01*	*(-24.07; -13.91)*	19.89 (132.30)	*-19.56*	*p* < *0.01*	*(-24.22; -14.91)*
*Monthly DMT costs (EUR)*	902.68 (406.47)	804.11 (418.64)	*-56.06*	*p* < *0.01*	*(-68.58; -43.80)*	854.19 (436.78)	*-58.06*	*p* < *0.01*	*(-69.18; -46.94)*	939.32 (470.68)	*-31.72*	*p* < *0.01*	*(-41.91; -21.53)*

Table shows the annualized hospitalization rate and monthly costs described as mean (standard deviation). Differences in outcomes between periods (Pre-lockdown (as reference), Lockdown, Pre-Vaccination and Vaccination) were explored using linear mixed models. *The analyses were adjusted for age, sex, and treatment duration

When compared with pre-COVID-19 period, AHR decreased during lockdown (Coeff = -0.64; 95%CI = -0.69, -0.59; *p* < 0.01), and remained significantly lower during pre-vaccination (Coeff = -0.37; 95%CI = -0.41, -0.33; *p* < 0.01), and vaccination periods (Coeff = -0.35; 95%CI = -0.39, -0.32; *p* < 0.01). Results were confirmed also after adjusting by adherence. After adjusting by Charlson Comorbidity index, when compared with pre-COVID-19 period, AHR was higher during lockdown (Coeff = 4.44; 95%CI = 3.98, 4.90; *p* < 0.01), pre-vaccination period (Coeff = 1.42; 95%CI = 1.30, 1.55; *p* < 0.01), and during vaccination period (Coeff = 0.31; 95%CI = 0.21, 0.39; *p* < 0.01), thus suggesting that comorbidities have increased the probability of hospitalization across all COVID-19 phases.

When compared with pre-COVID-19 period, MS AHR decreased during the lockdown (Coeff = -0.57; 95%CI = -0.62, -0.53; *p* < 0.01), and remained significantly lower during pre-vaccination (Coeff = -0.31; 95%CI = -0.36, -0.23; *p* < 0.01), and vaccination periods (Coeff = -0.29; 95%CI = -0.32, -0.26; *p* < 0.01). Results were confirmed also after adjusting by adherence. After adjusting by Charlson Comorbidity index, when compared with pre-COVID-19 period, MS AHR was higher during lockdown (Coeff = 2.91; 95%CI = 2.45, 3.37; *p* < 0.01) and pre-vaccination period (Coeff = 1.37; 95%CI = 1.25, 1.50; *p* < 0.01), but returned to pre-pandemic values during vaccination period (Coeff = 0.35; 95%CI = 0.26, 0.44; *p* < 0.01), thus confirming the effect of comorbidities on MS hospitalizations.

When compared with pre-COVID-19 period, costs for hospital admissions were lower during lockdown (Coeff = -40.19; 95%CI = -48.83, -31.56; *p* < 0.01), pre vaccination period (Coeff = -22.34; 95%CI = -30.08, -14.59; *p* < 0.01), and vaccination periods (Coeff = -22.25; 95%CI = -29.35, -15.16; *p* < 0.01). Results were confirmed also after adjusting by adherence.

When compared with pre-COVID-19 period, costs for MS hospital admissions were lower during lockdown (Coeff = -35.26; 95%CI = -40.93, -29.61; *p* < 0.01), pre vaccination (Coeff = -18.99; 95%CI = -24.07, -13.91; *p* < 0.01), and vaccination periods (Coeff = -19.56; 95%CI = -24.22, -14.91; *p* < 0.01). Results were confirmed also after adjusting by adherence.

When compared with pre-COVID-19 period, costs for DMTs were lower during lockdown (Coeff = -56.19; 95%CI = -68.58, -43.80; *p* < 0.01), pre vaccination (Coeff = -58.06; 95%CI = -69.18, -46.94; *p* < 0.01), and vaccination periods (Coeff = -31.72; 95%CI = -41.91, -21.53; *p* < 0.01). Results were confirmed also after adjusting by adherence. After adjusting by Charlson Comorbidity index, when compared with pre-COVID-19 period, costs for DMTs remained similar during lockdown (Coeff = -81.14; 95%CI = -167.77, 5.48; *p* = 0.06), but decreased during pre-vaccination (Coeff = -75.56; 95%CI = -99.04, -52.07; *p* < 0.01) and vaccination periods (Coeff = -22.06; 95%CI = -39.15, -4.96; *p* < 0.01).

## Discussion

Our population-based study showed changes in MS management during and following COVID-19 pandemic. We observed a decrease in all-cause and MS hospital admissions (and related costs) from lockdown and until recent time, thus suggesting a re-organization with de-centralized healthcare delivery. When including comorbidities in the statistical models, we found higher probability of hospitalization, when compared with pre-COVID-19, possibly reflecting increased awareness of comorbidities and related risks. This de-centralized model of care, however, might have resulted in reduced quality of care, with lower rates of adherence and lower DMT costs (e.g., as from the use of low/medium-efficacy DMTs). In keep with this, we observed a drop of new DMT prescriptions during the lockdown, which however quickly surged to pre-COVID-19 levels. Overall, our results suggest a significant impact of COVID-19 on MS management, but satisfactory recovery of the healthcare system in resuming activities after the great lockdown.

Healthcare utilization is high in the MS population, with up to 25.8% of the MS population being hospitalized annually, well above the rate of hospitalizations in the general population [15, 20, 21]. Hospitalizations are generally related to MS (e.g., new or worsening symptoms), its treatments (e.g., side effects), and chronic consequences of disability, such as urinary tract infections, which are the most common reason for hospitalization [10, 15, 22]. The observed declines in hospitalizations during and after COVID-19 may reflect changes in healthcare delivery, including the administration of therapy for relapses in outpatients or at home, rather than inpatient setting. However, a decentralized model of care might have been responsible for reduced rates of adherence, resulting from both limited access to usual medical services due to unavailability, and fear of SARS-CoV-2 infection [12, 23]. An assessment of the impact on long-term outcomes is needed [24].

Our study findings support that comorbidity is associated with a greater burden on healthcare systems. In fact, people with MS have higher rates of hospitalizations due to comorbidities (e.g., hypertension, diabetes, ischemic heart disease, chronic lung disease, depression, and bipolar disorder), compared with the general population [20]. This has further increased during and after COVID-19, thus suggesting PwMS have been further exposed to their frailty over the recent years.

Furthermore, consistent with other studies, we confirmed that comorbidities and their severity (i.e., Charlson comorbidity index) are strong predictors of hospitalization [20]. In particular, severe kidney disease, diabetes, ongoing chemotherapy, severe immunodeficiency, heart failure, and Down syndrome stand out as having a higher associated risk of hospitalization due to COVID-19 [25, 26].

Due to the possible effect of some DMTs on the frequency and severity of SARS-Cov-2 infection, the decision of whether to start, discontinue or continue on medications has been a critical issue for both patients and physicians. Most national neurological/MS societies and international working groups have advised against the use of highly effective DMTs amid the peak of COVID-19 pandemic and lockdown [27, 28]. This is fully reflected by our results. However, this is the first study to explore the recovery of the healthcare system after the pandemic, and we showed that delays in DMT use (including both new and switch prescriptions) quickly recovered to pre-COVID-19 levels.

Limitations of this study include the conduction in a single Italian Region, from which data is available at population level. However, COVID-19 has affected healthcare systems worldwide, and described impact and recovery are expected. Also, we did not assess the direct impact of COVID-19 infections that could have affected some outcomes (e.g., reduced adherence due to suspended or delayed treatment during active infection), which will grant further investigations. We have decided to focus on healthcare resource utilization only and did not include clinical data that would be available only for a subgroup of patients.

Moreover, there might be patterns of healthcare resource utilization that are associated with treatment decisions (e.g., patients less in contact with MS centers being less likely to use highly effective DMTs); this was not fully accounted in our study and warrants further investigations.

In conclusion, we have described profound changes of MS management following COVID-19 pandemic. While reduced hospitalization rates (and related costs) could be read as a proxy of improved care, there is the possibility of missed clinical events due to COVID-19 re-organization of healthcare delivery, as also suggested by reduced adherence. Similarly, the use of DMTs has plunged during the lockdown, but quickly came back to pre-COVID-19 levels, thus suggesting good recovery of the healthcare system and minimal effect on PwMS.
